# Sexual orientation differences in outpatient psychiatric treatment and antidepressant usage: evidence from a population-based study of siblings

**DOI:** 10.1007/s10654-018-0411-y

**Published:** 2018-05-15

**Authors:** Richard Bränström, Mark L. Hatzenbuehler, Petter Tinghög, John E. Pachankis

**Affiliations:** 1 Department of Social and Behavioral Sciences, Yale School of Public Health, 60 College St., Suite 316, New Haven, CT 06520 USA; 2 Department of Clinical Neuroscience, Karolinska Institutet, Stockholm, Sweden; 3 Department of Sociomedical Sciences, Columbia University, New York, NY USA; 4 Department of Public Health and Medicine, Red Cross University College, Stockholm, Sweden

**Keywords:** Sexual minorities, Depression, Anxiety, Psychiatric disorders, Sibling design

## Abstract

In the past two decades, population-based health surveys have begun including measures of sexual orientation, permitting estimates of sexual orientation disparities in psychiatric morbidity and differences in treatment utilization. The present study takes advantage of the high-quality, comprehensive nationwide health registry data available in Sweden to examine whether psychiatric outpatient treatment for various diagnoses and antidepressant medication usage are greater in sexual minority individuals compared to their siblings. A longitudinal cohort study design was used with a representative random population-based sample in Stockholm, Sweden. Registry-based health record data on all specialized outpatient health care visits and prescription drug use was linked to a sample of 1154 sexual minority individuals from the Stockholm Public Health Cohort and their siblings. The main outcomes were treatment due to psychiatric diagnoses retrieved from nationwide registry-based health records. In analyses accounting for dependency between siblings, gay men/lesbians had a greater likelihood of being treated for mood disorder [adjusted odds ratio (AOR) 1.77; 99% confidence intervals (CI) 1.00, 3.16] and being prescribed antidepressants (AOR 1.51; 99% CI 1.10, 2.07) compared to their siblings. Further, bisexual individuals had a greater likelihood of any outpatient psychiatric treatment (AOR 1.69; 99% CI 1.17, 2.45) and being prescribed antidepressants (AOR 1.48; 99% CI 1.07, 2.05) as well as a greater likelihood of being treated for a mood disorder (AOR 1.98; 99% CI 1.33, 2.95) compared to their siblings. No difference in anxiety or substance use disorder treatment was found between any sexual minority subgroup and their siblings. The potential role of familial confounding in psychiatric disorder treatment was not supported for more than half of the outcomes that were examined. Results suggest that sexual minority individuals are significantly more likely to be treated for certain psychiatric disorders compared to their siblings. Future research is needed to understand mechanisms other than familial factors that might cause the substantial treatment differences based on sexual orientation reported here.

**Electronic supplementary material:**

The online version of this article (10.1007/s10654-018-0411-y) contains supplementary material, which is available to authorized users.

## Introduction

In the past two decades, population-based health surveys have begun including measures of sexual orientation, permitting estimates of sexual orientation disparities in psychiatric morbidity and differences in treatment utilization. These studies have shown that sexual minority individuals (e.g., those who identify as lesbian, gay, or bisexual; engage in same-sex sexual behavior; or report persistent attractions to individuals of the same sex) are at significantly greater risk of mood, anxiety, and substance use disorders and are more likely to seek treatment for those disorders than heterosexuals [1–5]. Previous studies have suggested that sexual minorities might utilize mental health treatment more often than heterosexuals for several reasons, including their greater risk of mental health problems, unique developmental stressors such as adjusting to a sexual minority identity, and positive community norms toward mental health treatments [6–8].

Our current study advances this literature on sexual orientation differences in mental health treatment utilization in several ways. First, prior studies have relied on self-reports of treatment utilization [9–13], which may be subject to reporting bias. We use objective measures of health care utilization obtained from comprehensive medical registries in Sweden, thereby permitting a more accurate test of sexual orientation differences in treatment utilization than heretofore possible. Second, to increase sample size, population-based studies have typically combined sexual orientation identity subgroups, including gay men/lesbians and bisexuals [14], which may obscure unique patterns of sexual orientation differences in treatment utilization for psychiatric disorders. Our study uses a large population-based sample of sexual minorities that permits the examination of these potential subgroup differences. Third, existing studies have compared mental health treatment utilization outcomes between sexual minorities and unrelated heterosexuals [9–13]. While this approach is certainly warranted, researchers have also noted that the siblings of sexual minorities provide another strong comparison group [19, 20]. Sibling designs are particularly relevant for studies of health-care seeking, given that family members resemble one another in their utilization of both general and acute health care [15–17], with 20–30% of treatment-seeking behaviors being explained by within-family influences [15, 16]. These findings regarding family influences on treatment utilization are likely due to shared socialization influences within families, such as norms around treatment-seeking for healthcare problems [17]. Although studies have compared the mental health of sexual minority individuals to that of their heterosexual siblings [18–20], no study has utilized a sibling design to examine odds of outpatient psychiatric treatment as a function of sexual minority identity. To address this gap, the present study takes advantage of the high-quality, comprehensive nationwide health registry data available in Sweden linked to a large cohort of sexual minority individuals and their siblings. This unique data structure permitted us to examine whether outpatient psychiatric treatment for various disorders and antidepressant medication usage are greater in sexual minority individuals compared to their siblings.

## Method

### Participants and general procedure

Participants were drawn from the Stockholm Public Health Cohort (SPHC), a prospective study managed by the Stockholm County Council [21]. In 2002, 2006, and 2010, population-based health surveys were conducted in random samples of the population among individuals in Stockholm County aged 18 years and older. At each year of assessment, approximately 50,000 individuals were invited to participate, and those already included in the cohort were asked to respond to a follow-up questionnaire (i.e., in 2006 and 2010). The total response rate was 62% in 2002, 61% in 2006, and 56% in 2010. In the 2010 survey, one question regarding sexual orientation identity was included: “How do you define your sexual orientation?” with the response categories “heterosexual,” “homosexual,” “bisexual,” and “uncertain.”

A total of 66,604 (92.0%) individuals responded that they identified as heterosexual, 848 (1.2%) as gay/lesbian, and 806 (1.1%) as bisexual. The proportion of sexual minority individuals in the current sample corresponds to the proportion reported in other population-based studies in Sweden. Specifically, national population-based studies conducted between 2005 and 2015 have found the proportion of respondents reporting a sexual minority status ranges from 2.2 to 2.6% [22]. We excluded 394 (1.3%) individuals who responded that they were uncertain of their sexual orientation, as previous studies have shown that this group often consists of a heterogeneous mix of respondents in terms of sexual identity [23] and that the majority of people who choose such responses in population surveys do so because they did not understand the question [24]. Those who did not respond to the sexual orientation question (n = 1160; 3.8%) were also excluded from the study. Analyses showed that this group was more likely to be older, female, single, and born outside of Sweden, and to have lower education and income, than those who responded (all *P* < 0.001). The sexual minority sample contained the 1154 participants in the cohort who self-identified as gay, lesbian, or bisexual and who had at least one sibling.

Sexual minority men and women were compared to two groups in the statistical analyses. For the primary analyses, the sexual minority group was compared to their full siblings identified using the comprehensive Swedish multi-generation registry [25]. The multi-generation registry is a part of the Total Population Registry in Sweden and contains information about kinship and adoption history for all individuals living in Sweden. Linkage between individuals is possible through use of personal identification numbers. The registry is updated yearly with new information added regarding migration, deaths, births, and adoptions. A total of 1643 full siblings were identified, an average of 1.4 siblings per sexual minority individual. Because the siblings did not complete the questionnaires administered to the SPHC, most sociodemographic information, including sexual orientation, was unavailable for this group. As the sexual orientation of this primary comparison group was not known, and in order to estimate the proportion of the sexual orientation disparity in mental health treatment that can be explained by shared factors within families, we conducted an additional comparison among all self-identified heterosexuals in the SPHC with at least one sibling in the registry. A total of 48,454 individuals (i.e., 72.7% of all self-identified heterosexuals) had at least one sibling, and constituted the unrelated self-identified heterosexual control group used in our secondary analyses.

### Measures

All specialized outpatient health care visits for individuals in the cohort were obtained from comprehensive nationwide registries between January 1, 2005 to December 31, 2011 and linked to the SPHC using the personal identification numbers available in Sweden. Each psychiatric visit had been coded by the treating physician with a primary diagnosis from the International Statistical Classification of Diseases and Related Health Problems version 10 (ICD-10) [26] and up to 20 supplementary ICD-10 diagnostic codes. Primary and supplementary diagnostic codes were used in these analyses. All individuals were classified as having received treatment for any or no diagnosis during the specified period based on the presence of psychiatric diagnostic codes (i.e., ICD-10: F00–F95). Psychiatric diagnoses were subsequently categorized into treatment for any: mood disorder (ICD-10: F30–F39), anxiety disorder (ICD-10: F40–F42), or substance use disorder (ICD-10: F10–F19). In addition, more specific categorizations were made for major depressive disorder (ICD-10: F32, F33) and generalized anxiety disorder (ICD-10: F41). In addition, the total number of medical outpatient visits for any cause, both somatic and psychiatric, was calculated for each individual and was included as a covariate in the analyses in order to minimize the likelihood that any observed differences were due to differences in engagement with the healthcare system.

Use of prescription medication was obtained from the Swedish Prescribed Drug Registry, which contains information regarding all prescribed and purchased medication nationwide for all individuals in the cohort from July 1, 2005 to December 31, 2012. Antidepressant medication was classified according to the Anatomical Therapeutic Chemical Classification system (code N06A). Individuals were categorized into ‘any use’ vs. ‘no use’ based on date of purchase of prescribed antidepressant medication.

Sociodemographic information was linked to each respondent in the Stockholm Public Health Cohort from national registries by using personal identification numbers. Information derived from the registries included age (in years), gender (legal gender, i.e., man or woman), number of siblings, education (university education vs. no university education), income (monthly individual income in Swedish kronor), and country of birth (born in Sweden; born in another European Union country; born outside of the European Union). In addition to the sociodemographic variables taken from the registries, information about relationship status was collected using Stockholm Public Health Cohort survey data regarding whether the respondent shared a household with a partner (yes/no).

### Statistical analysis

The primary analyses compared outpatient treatment for various psychiatric disorders and antidepressant usage between sexual minority individuals and their own siblings. Generalized estimating equations (GEE) with exchangeable covariance structure were used to account for dependency within sibling pairs and groups of siblings. These analyses were adjusted for age, gender, number of siblings in the family, and total number of medical outpatient visits for any cause.

For the secondary analyses, logistic regression was used to examine sexual orientation differences in outpatient treatment for psychiatric disorders and antidepressant usage between sexual minority individuals and self-identified heterosexuals with at least one sibling. These analyses were adjusted for age, gender, number of siblings, and total number of medical outpatient visits for any cause.

To estimate potential shared familial influences, we calculated the percent reduction in the difference when sexual minority individuals were compared to their siblings versus when they were compared to unrelated heterosexuals with siblings. Specifically, we subtracted the difference derived from models comparing sexual minority individuals to siblings of sexual minority individuals from the difference derived from models comparing sexual minority individuals to unrelated heterosexuals. These analyses allowed us to examine the potential presence of unmeasured familial confounding in explaining the association between sexual identity and outpatient psychiatric treatment.

In addition to the main analyses, three sets of sensitivity analyses were conducted. First, the comparisons between the sexual minority sample and their siblings were restricted to include only siblings 18 years of age or older (the youngest age in the sexual minority sample). This enabled us to determine whether exclusion of younger siblings, who have had less time to develop a potential psychiatric disorder, affected the results. Second, because no significant gender-by-sexual orientation interaction effects were found in the primary and secondary analyses, and because of the low number of outpatient visits for some psychiatric disorders, we did not stratify our primary and secondary analyses by gender. However, as the gender-by-sexual orientation interaction for outpatient treatment for any mental health diagnosis among bisexual individuals approached significance, we explored the results for bisexual individuals stratified by gender in an additional set of sensitivity analyses. Third, we tested the robustness of our findings in the secondary analyses (i.e., comparing sexual minorities and un-related self-identified heterosexuals) by controlling for four additional socio-demographic variables that were not available for the sibling control sample used in the primary analyses, including education, income, country of birth, and relationship status.

The level for statistical significance in regression models was set at α = 0.01 due to the large number of planned statistical tests. Data analyses were conducted using SPSS, version 24.

## Results

Table 1 presents the age, gender, and distribution of outpatient visits for psychiatric disorders and antidepressant use among sexual minority individuals in the SPHC, siblings of sexual minority individuals, and self-identified heterosexuals in the SPHC with at least one sibling. Bisexual individuals were significantly younger than gay men/lesbians (*t *= 8.52, *P* < 0.001) and self-identified heterosexual individuals (*t *= 17.46, *P* < 0.001), and gay men/lesbians were younger than self-identified heterosexual individuals (*t *= 6.24, *P* < 0.001).

**Table 1:** Age, number of siblings, outpatient visits for psychiatric disorders, and antidepressant usage by sexual orientation and relatedness

	Gay/lesbian n = 582	Bisexual n = 572	Siblings of gay, lesbian, or bisexual individuals n = 1643	Unrelated heterosexuals with siblings n = 48,454
	Mean (SD)	Mean (SD)	Mean (SD)	Mean (SD)
Age	45.6 (13.5)	38.7 (15.0)	43.3 (15.7)	49.2 (14.8)
Gender				
Men	339 (58.2)	178 (31.1)	820 (49.9)	21,385 (44.1)
Women	243 (41.8)	394 (68.9)	823 (50.1)	27,069 (55.9)

	n (%)	n (%)	n (%)	n (%)
Psychiatric outpatient visits (2005–2011)				
Treatment for any mental health diagnosis	70 (12.0)	115 (20.1)	157 (9.6)	3473 (7.2)
Any mood disorder	38 (6.5)	58 (10.1)	60 (3.7)	1438 (3.0)
Major depressive disorder	35 (6.0)	49 (8.6)	54 (3.3)	1225 (2.5)
Anxiety disorder	21 (3.6)	37 (6.5)	61 (3.7)	1115 (2.3)
Generalized anxiety disorder	13 (2.2)	15 (2.9)	34 (2.1)	449 (0.9)
Substance use disorder	21 (3.6)	26 (4.5)	42 (2.6)	708 (1.5)
Antidepressant treatment (2005–2012)				
Any antidepressant use	140 (24.1)	152 (26.6)	274 (16.7)	7661 (15.8)

### Outpatient treatment for psychiatric disorders and antidepressant use among sexual minority individuals and their siblings

Between 2005 and 2011, 12.0% of gay men/lesbians, 20.1% of bisexual individuals, and 9.6% of their siblings had at least one psychiatric outpatient health care visit. The difference in mean number of visits between the sexual minority individuals and their siblings was statistically significant (*F *= 29.00, *P* < 0.001). During the study period, a significantly higher proportion of sexual minority individuals (24.1% of gay men/lesbians and 26.6% of bisexual individuals) had used any antidepressant compared to 16.7% of their siblings (*χ*^2^ = 33.19, *P* < 0.001).

Table 2 presents the results of the primary GEE analyses showing differences in outpatient treatment for psychiatric disorders and antidepressant use between sexual minority individuals and their siblings. Gay men/lesbians had an elevated likelihood of being treated for mood disorder and being prescribed antidepressants compared to their siblings. Bisexual individuals had an elevated likelihood of being treated for any psychiatric diagnosis and mood disorder, and of being prescribed antidepressants, compared to their siblings. In terms of specific mood disorder diagnoses, gay men, lesbians, and bisexual individuals had an elevated likelihood of being treated for major depressive disorder relative to their siblings.

**Table 2:** Sexual orientation differences in outpatient treatment for psychiatric disorders and antidepressant usage comparing gay, lesbian, and bisexual individuals to their siblings and to unrelated heterosexual individuals

Variable	Primary analysis: differences in outpatient treatment for psychiatric disorders and antidepressant usage between sexual minority individuals and their siblings (reference)				Secondary analysis: differences in outpatient treatment for psychiatric disorders and antidepressant usage between sexual minority individuals and unrelated heterosexuals with at least one sibling (reference)			
	Gay men/lesbians		Bisexual men and women		Gay men/lesbians		Bisexual men and women	
	AOR^a^	99% CI	AOR^a^	99% CI	AOR^b^	99% CI	AOR^b^	99% CI
Psychiatric outpatient visits 2005–2011								
Treatment for any mental health diagnosis	1.22	0.80, 1.85	1.69**	1.17, 2.45	1.64**	1.16, 2.31	2.49**	1.87, 3.31
Any mood disorder	1.77*	1.00, 3.16	1.98**	1.33, 2.95	2.19**	1.38, 3.45	2.62**	1.80, 3.84
Major depressive disorder	1.86*	1.03, 3.35	1.87*	1.08, 3.22	2.35**	1.47, 3.78	2.50**	1.66, 3.75
Any anxiety disorder	0.91	0.45, 1.86	1.11	0.61, 2.02	1.40	0.76, 2.56	1.82**	1.15, 2.88
Generalized anxiety disorder	1.04	0.41, 2.62	0.77	0.32, 1.89	2.28*	1.06, 4.90	1.94	0.97, 3.89
Substance use disorder	1.25	0.56, 2.77	1.71	0.85, 3.42	2.16**	1.19, 3.93	3.35**	1.97, 5.71
Antidepressant treatment 2005–2012								
Any antidepressant use	1.51**	1.10, 2.07	1.48*	1.07, 2.05	1.91**	1.47, 2.48	1.82**	1.41, 2.35

*AOR* adjusted odds ratios, *CI* confidence interval

^a^Generalized estimation equation analyses adjusted for age, gender, number of siblings, and total number of health care visits for any cause between 2005 and 2011

^b^Logistic regression analyses adjusted for age, gender, number of siblings, and total number of health care visits for any cause between 2005 and 2011

*P < 0.01; **P < 0.001

### The role of unmeasured familial confounding in explaining sexual orientation differences in outpatient treatment for psychiatric disorders and antidepressant use

**Fig. 1:**
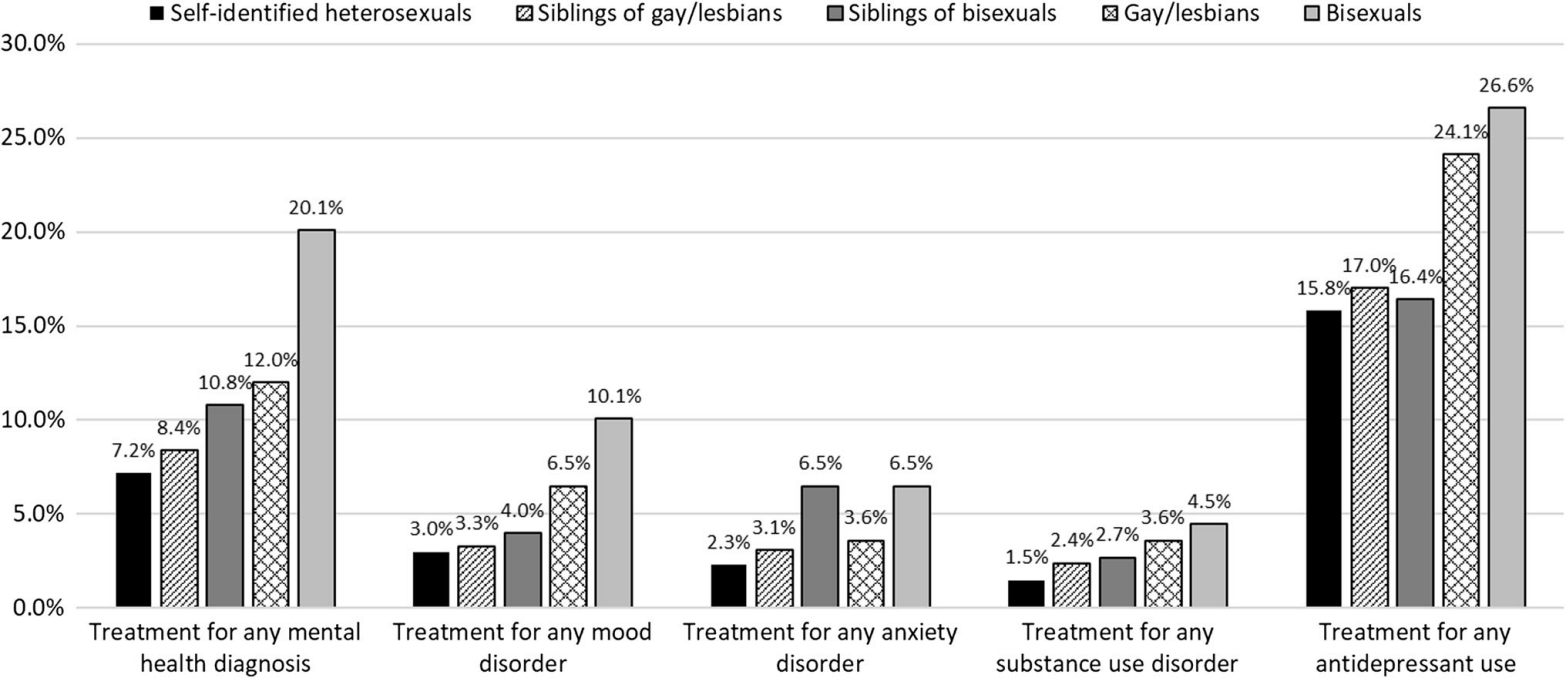
Prevalence of treatment for psychiatric disorders and use of antidepressants among sexual minorities, their siblings, and unrelated heterosexuals

To examine if a portion of the sexual orientation-based differences in outpatient psychiatric treatment could be explained by shared factors within families, we compared the size of the difference when sexual minority individuals were compared to their siblings versus when they were compared to unrelated heterosexuals (Table 2; Fig. 1). The odds ratios for the sexual orientation-based difference in being treated for certain psychiatric disorders were larger when sexual minority individuals were compared to unrelated heterosexuals versus when they were compared to their siblings. Specifically, among bisexual individuals, the odds ratio was reduced by 39% for treatment for any anxiety disorder diagnosis and by 49% for treatment for substance use disorders; furthermore, the sexual orientation disparity was no longer evident for treatment for these disorders. Among gay men and lesbians, the odds ratio was reduced by 54% for treatment for generalized anxiety disorder and by 42% for treatment for substance use disorder; the sexual orientation disparity was no longer evident for treatment for either disorder.

### Sensitivity analyses

We ran three sets of sensitivity analyses. First, we reran the primary analyses using only siblings 18 years and older as the comparison, which showed an identical pattern of results (Supplementary online eTable 1).

Second, in gender-stratified models among bisexual individuals (Supplementary online eTable 2), there were no statistically significant differences for any outcomes between bisexual men and their siblings. However, bisexual women had an elevated likelihood of treatment for any mental health diagnosis (AOR 1.90; 99% CI 1.20, 3.03), major depressive disorder (AOR 1.98; 99% CI 1.04, 3.77), and antidepressant use (AOR 1.63; 99% CI 1.09, 2.43) relative to their siblings.

Third, for the sample used in the secondary analyses (i.e., sexual minorities and non-related self-identified heterosexuals), we performed an additional set of logistic regression analyses adjusted for all available sociodemographic variables, including age, gender, number of siblings, education, income, country of birth, relationship status, and total number of medical outpatient visits for any cause. In these adjusted analyses, the odds ratios for differences between gay, lesbian, and bisexual individuals compared to heterosexuals were slightly reduced but remained significant for treatment for nearly all mental health diagnosis; however, the difference in treatment for generalized anxiety disorder between gay men and lesbians compared to heterosexuals became non-significant (Supplementary online eTable 3).

## Discussion

This study presents evidence of sexual orientation differences in outpatient psychiatric treatment and antidepressant use in a population-based sample of sexual minority individuals and their siblings. This is the first known study to examine treatment for psychiatric disorders derived from clinical settings in a population-based sample of sexual minority adults and their siblings, thereby overcoming limitations of self-reported mental health treatment and non-representative sampling.

Gay men/lesbians had a greater likelihood of being treated for a mood disorder and were more likely to use antidepressants compared to their siblings. Further, bisexual individuals had a greater likelihood of any outpatient psychiatric treatment and antidepressant use, and a greater likelihood of being treated for mood disorder, compared to their siblings. In terms of treatment for specific mood disorder diagnoses, all sexual minorities had greater likelihood of being treated for major depressive disorder compared to their siblings.

Because the sexual orientation of siblings could not be ascertained, we also compared sexual minority men and women to self-identified heterosexuals in the SPHC with at least one sibling. Using this sample of unrelated known-heterosexual individuals, we found that gay men/lesbians continued to have a higher rate of treatment for mood disorders and antidepressant use, but also showed a greater likelihood of outpatient psychiatric treatment for any disorder, for generalized anxiety disorder, and for substance use disorder, compared to heterosexuals. Further, bisexual individuals continued to show greater likelihood of treatment for all psychiatric disorders, including major depressive disorder, any anxiety disorder, and substance use disorder, in addition to antidepressant use, compared to heterosexuals.

The present study extends extant findings in several important ways. First, previous population-based studies that have used self-report assessment of mental health visits have similarly documented that sexual minorities utilize mental health treatment at higher rates than heterosexuals [5]. The current study extends these findings, using objective measures of health care utilization obtained from medical registries.

Second, the use of both a complete set of siblings as a comparison group, including all living full siblings of the sexual minority individuals, as well as unrelated heterosexuals, allowed us to examine the potential role of familial confounding in partially explaining sexual orientation differences in treatment for psychiatric disorders. We found a potential role of familial confounding in more than a third of the examined treatment differences, consistent with prior work showing that treatment-seeking behaviors can be partially explained by within-family influences [15, 16]. Specifically, the significantly higher odds of treatment for any mental health problem, generalized anxiety disorder, and substance use disorder for gay men/lesbians when compared to unrelated heterosexuals became non-significant when compared to their siblings. Further, the odds of treatment for any anxiety disorders and substance use disorder for bisexual individuals when compared to unrelated heterosexual individuals were substantially reduced and became non-significant when compared to their siblings. These findings suggest that unmeasured factors within families, such as social norms related to mental health treatment-seeking, might at least partially explain associations between sexual orientation and treatment for these disorders. Indeed, family members have similar utilization patterns of both general and acute health care, perhaps due to shared early socialization influences [15–17]. The current study extends this possibility to psychiatric treatment utilization among sexual minorities and their siblings.

However, the potential role of familial confounding was not supported for more than half of the differences in psychiatric disorder treatment found here, suggesting that unique experiences related to sexual orientation are contributing to the observed differences in these treatment outcomes. Future research is needed to understand the sexual orientation-specific mechanisms that might cause these treatment differences based on sexual orientation. For instance, the potential causal role of sexual minority-specific stress in outpatient psychiatric treatment warrants greater attention, as existing research suggests that such stress plays an important role in explaining sexual orientation disparities in self-reported mental health [27–29] and that sexual minorities are more likely than heterosexuals to seek mental health treatment [30]. Because research shows that sexual minority men are more likely than heterosexual men to seek mental health care even outside the context of mental health impairment [30], future research should also determine what factors beyond minority stress and mental health impairment, such as unique experiences and cultural norms (e.g., support for sexual identity development), might explain sexual minorities’ increased utilization of psychiatric treatments [31]. Further, because recent research shows that improvements in the structural climate in Sweden in the past several years (e.g., removal of legislative discrimination toward sexual minorities) are associated with concomitant decreases in the sexual orientation disparity in psychological distress during that time period [22], future research is needed to explore why most differences in treatment utilization, other than for anxiety disorders, persist here. The difference between the present study and recent findings of reduced disparities in psychological distress in Sweden could be explained by differences in the included time period, outcome measurements, or other factors.

Third, because previous studies of sexual orientation disparities in mental health that have used siblings as controls either did not stratify analyses by sexual identity [32] or operationalized sexual orientation in terms of behavior [33] or attraction [34], our study possessed the unique ability to determine the specificity of results to individuals who identify as bisexual as compared to gay or lesbian. Overall, we found that bisexual individuals experienced similar differences in outpatient psychiatric treatment as gay men and lesbians, although we found some differences in patterns of potential familial confounding depending on sexual identity. Whereas for gay men and lesbians we found that potential familial confounding was limited to treatment for any psychiatric disorder, generalized anxiety disorder, and substance use disorders, for bisexuals, we found that potential familial confounding was limited to treatment for any anxiety disorder and substance use disorder.

While this study possesses several notable strengths in sampling (i.e., population-based), assessment of mental health treatment (i.e., derived from treatment registries), and comparison groups (i.e., both siblings and self-identified heterosexuals), results must be interpreted in light of several limitations. First, the universal health coverage in Sweden likely limits the effect of different access to health care treatment between sexual orientation groups, and thus our results are likely unique to this context.

Second, clinician-derived psychiatric diagnosis is subject to clinical decision-making in a treatment context. Given the lack of objective disease markers in most psychiatric presentations [35], clinical diagnosis remains subject to bias. While the nature and direction of this bias as it applies to sexual orientation is unknown, one particularly important potential bias in need of future study involves clinicians’ tendency to understand some aspects of sexual identity development (e.g., identity confusion) as psychiatric symptoms rather than as normative features of sexual identity development [36].

Third, some of the psychiatric disorders analyzed in the current study were relatively rare in this sample. The relatively small sample sizes for treatment for certain diagnoses, such as generalized anxiety disorder and substance use disorders, increases the risk of type 2 error. It is possible that some of the non-significant estimates of sexual orientation differences in treatment for these diagnoses would have been significant with a larger sample, and the results should be interpreted in light of this limitation. Fourth, this study relied on sexual identity as a measure of sexual orientation, precluding examination of whether these findings extend across all dimensions of sexual orientation (e.g., sexual behavior, patterns of attraction). Fifth, the sexual identity of the siblings in our sample was not assessed and it is possible that some of the siblings were also sexual minority individuals. However, due to the low proportion of sexual minorities in the general population, we consider the influence of this possible misclassification on our overall results to be low. Lastly, even with the strong methodology provided by a sibling comparison study, it is not possible to completely account for the complexity of familial influence with this design, given that all family members have a unique experience of that family [37].

In conclusion, this study takes advantage of the high-quality health registry data available in Sweden to conduct the first examination of sexual orientation differences in outpatient psychiatric treatment and antidepressant use in a population-based sample with sibling comparisons. Results suggest that gay men, lesbians, and bisexual women are significantly more likely to receive treatment for mood disorders compared to their siblings, with potential evidence of some shared etiologic influences between sexual minority status and psychiatric morbidity relating to anxiety and substance use disorders. Future studies can extend these findings by continuing to investigate the etiological determinants and experiences of psychiatric treatment-seeking capable of explaining the sexual orientation differences in outpatient psychiatric treatment and antidepressant use found here.

## Electronic supplementary material

Below is the link to the electronic supplementary material.

Supplementary material 1 (DOCX 36 kb)
