# Supplementary material for: Sexual orientation differences in outpatient psychiatric treatment and antidepressant usage: evidence from a population-based study of siblings
Source: Eur J Epidemiol. 2018 May 15;33(6):591–9. doi: 10.1007/s10654-018-0411-y (PMC5995973; doi:10.1007/s10654-018-0411-y)
Supplement: Supplementary file 1 — Supplementary material 1 (DOCX 36 kb) [file 10654_2018_411_MOESM1_ESM.docx]

Supplementary online tables

**eTable 1** Sexual orientation differences in outpatient treatment for psychiatric disorders and antidepressant usage comparing sexual minority individuals to their siblings ages 18 years or older

**eTable 2:** Sexual orientation differences in outpatient treatment for psychiatric disorders and antidepressant usage comparing bisexual men and women to their siblings

**eTable 3:** Sexual orientation differences in outpatient treatment for psychiatric disorders and antidepressant usage comparing gay, lesbian, and bisexual individuals to unrelated heterosexual individuals adjusted for age, gender, education, income, country of birth, relationship status, number of siblings, and total number of health care visits for any cause between 2005 and 2011

**eTable 1:** Sexual orientation differences in outpatient psychiatric treatment for given disorders and antidepressant usage comparing sexual minority individuals to their siblings ages 18 years or older

|  | | | **Gay Men/Lesbian women** | |  | **Bisexual individuals** | |
| --- | --- | --- | --- | --- | --- | --- | --- |
|  | | | AOR | 99% CI |  | AOR | 99% CI |
| Psychiatric outpatient visits 2005 to 2011: | | |  |  |  |  |  |
| **Treatment for any mental health diagnosis** | | | 1.19 | 0.78, 1.81 |  | 1.65** | 1.13, 2.40 |
|  | | |  |  |  |  |  |
| **Any mood disorder** | | | 1.70 | 0.95, 3.02 |  | 1.86* | 1.09, 3.16 |
| Major depressive disorder | | 1.78* | 1.00, 3.21 |  | 1.76* | 1.01, 3.06 |  |
|  | |  |  |  |  |  |  |
| **Any anxiety disorder** | | | 0.87 | 0.43, 1.76 |  | 1.03 | 0.56, 1.90 |
| Generalized anxiety disorder | | 1.01 | 0.40, 2.53 |  | 0.73 | 0.30, 1.78 |  |
|  | |  |  |  |  |  |  |
| **Substance use disorder** | | | 1.23 | 0.55, 2.73 |  | 1.68 | 0.84, 3.37 |
|  | | |  |  |  |  |  |
| Antidepressant treatment 2005 to 2012: | | |  |  |  |  |  |
| **Any antidepressant use** | 1.49** | 1.09, 2.04 |  | 1.45* | 1.05, 2.02 |  |  |
|  | | |  |  |  |  |  |
| * = P < .01; ** = P < .001; AOR = adjusted odds ratios; CI = confidence interval.  All analyses were conducted using generalized estimating equations and were adjusted for age, gender, number of siblings, and total number of health care visits for any cause between 2005 and 2011. | | | | | | | |

**eTable 2:** Sexual orientation differences in outpatient psychiatric treatment for given disorders and antidepressant usage comparing bisexual men and women to their siblings

|  | | | **Bisexual men** | |  | **Bisexual women** | | |
| --- | --- | --- | --- | --- | --- | --- | --- | --- |
| **Variable** | | | AOR | 99% CI |  | AOR | 99% CI | |
| Psychiatric outpatient visits 2005 to 2011: | | |  |  |  |  |  | |
| **Treatment for any mental health diagnosis** | | | 1.22 | 0.59, 2.50 |  | 1.90** | 1.20, 3.03 | |
|  | | |  |  |  |  |  | |
| **Any mood disorder** | | | 1.45 | 0.47, 4.48 |  | 2.12* | 1.12, 4.01 | |
| Major depressive disorder | | 1.20 | 0.32, 4.51 |  | 1.98* | 1.04, 3.77 | |  |
|  | |  |  |  |  |  | |  |
| **Any anxiety disorder** | | | 1.30 | 0.36, 4.74 |  | 0.95 | 0.48, 1.90 | |
| Generalized anxiety disorder | | 0.42 | 0.03, 5.98 |  | 0.68 | 0.26, 3.13 | |  |
|  | |  |  |  |  |  | |  |
| **Substance use disorder** | | | 1.19 | 0.38, 3.72 |  | 1.91 | 0.62, 5.93 | |
|  | | |  |  |  |  |  | |
| Antidepressant treatment 2005 to 2012: | | |  |  |  |  |  | |
| **Any antidepressant use** | 1.10 | 0.58, 2.09 |  | 1.63* | 1.09, 2.43 | |  |  |
|  | | |  |  |  |  |  | |
| * = P < .01; ** = P < .001; AOR = adjusted odds ratios; CI = confidence interval.  All analyses were conducted using generalized estimating equations and were adjusted for age, gender, number of siblings, and total number of health care visits for any cause between 2005 and 2011. | | | | | | | |  |

**eTable 3:** Sexual orientation differences in outpatient psychiatric treatment for given disorders and antidepressant usage comparing gay, lesbian, and bisexual individuals to unrelated heterosexual individuals adjusted for age, gender, education, income, country of birth, relationship status, number of siblings, and total number of health care visits for any cause between 2005 and 2011

|  | | | **Gay Men/Lesbian women** | |  | **Bisexual individuals** | |
| --- | --- | --- | --- | --- | --- | --- | --- |
|  | | | AOR ^a^ | 99% CI |  | AOR ^a^ | 99% CI |
| Psychiatric outpatient visits 2005 to 2011: | | |  |  |  |  |  |
| **Treatment for any mental health diagnosis** | | | 1.53* | 1.07, 2.17 |  | 2.13** | 1.59, 2.85 |
|  | | |  |  |  |  |  |
| **Any mood disorder** | | | 2.04** | 1.29, 3.22 |  | 2.22** | 1.51, 3.28 |
| Major depressive disorder | | 2.21** | 1.37, 3.55 |  | 2.16** | 1.43, 3.27 |  |
|  | |  |  |  |  |  |  |
| **Any anxiety disorder** | | | 1.38 | 0.75, 2.52 |  | 1.63* | 1.03, 2.59 |
| Generalized anxiety disorder | | 2.08 | 0.96, 4.46 |  | 1.85 | 0.92, 3.71 |  |
|  | |  |  |  |  |  |  |
| **Substance use disorder** | | | 1.98* | 1.07, 3.65 |  | 2.84** | 1.65, 4.86 |
|  | | |  |  |  |  |  |
| Antidepressant treatment 2005 to 2012: | | |  |  |  |  |  |
| **Any antidepressant use** | 1.84** | 1.41, 2.39 |  | 1.63** | 1.26, 2.12 |  |  |
|  | | |  |  |  |  |  |
| * = P < .01; ** = P < .001; AOR = adjusted odds ratios; CI = confidence interval.  ^a^ Logistic regression analyses adjusted for age, gender, number of siblings, education, income, country of birth, relationship status, and total number of health care visits for any cause between 2005 and 2011. | | | | | | | |
